# Changes in Cerebrospinal Fluid, Liver and Intima-media-thickness Biomarkers in Patients with HIV-associated Neurocognitive Disorders Randomized to a Less Neurotoxic Treatment Regimen

**DOI:** 10.1007/s11481-023-10086-7

**Published:** 2023-10-31

**Authors:** Giacomo Stroffolini, Alessandro Lazzaro, Ambra Barco, Veronica Pirriatore, Daniela Vai, Claudia Giaccone, Marco Nigra, Cristiana Atzori, Mattia Trunfio, Stefano Bonora, Giovanni Di Perri G, Andrea Calcagno

**Affiliations:** 1 Department of Medical Sciences, Infectious Diseases Unit, University of Turin, Turin, Italy; 2 Department of Infectious-Tropical Diseases and Microbiology, IRCCS Sacro Cuore Don Calabria Hospital, Negrar, Verona, Italy; 3 Department of Public Health and Infectious Diseases, Sapienza University of Rome, Rome, Italy; 4 Department of Infectious Diseases, Novara Hospital, Novara, Italy; 5 Maria Vittoria Hospital, Unit of Neurology, Asl Città di Torino, Turin, Italy; 6 San Giovanni Bosco Hospital, Laboratory, Asl Città di Torino, Turin, Italy; 7 Maria Vittoria Hospital, Laboratory, Asl Città di Torino, Turin, Italy

**Keywords:** HAND, PLWH, Neuromarkers, Non-invasive tools, Neurovirology

## Abstract

The prevalence of neurocognitive impairment in people living with HIV is estimated between 30 and 50%. The pathogenesis of HIV-associated neurocognitive disorders is complex and multifactorial. Aim of the study was to measure the change in CSF biomarkers, Fibroscan and IMT measurements in PLWH with HAND randomized to a less neurotoxic regimen, or continuing their treatment. Adult patients with HAND were screened and enrolled if presenting no major resistance associated mutations, no HIV viral replication, not on efavirenz or darunavir, with R5-tropic HIV and without major confounding conditions. Lumbar puncture, IMT and Fibroscan measurements were performed. After 1:1 randomization to a less neurotoxic regimen consisting of darunavir/cobicistat plus emtricitabine plus maraviroc, or mantaining actual care, tests were repeated after 24 weeks: CSF biomarkes (HIV RNA, tau, p-tau, Beta-amyloid_1-42_, S100Beta and neopterin) were included. Non-parametric tests (Mann–Whitney and Wilcoxon’s) were used. 28 participants completed the study. Male and European ancestry were prevalent; median age was 55 years (51–60). All patients were virally suppressed; median CD4 + count was 626 cell/uL (469–772). Baseline characteristics were similar between the study arms. A significant decrease in CSF p-tau and an increase in CSF neopterin and NFL were observed. We observed a significant reduction in liver stiffness at W24. Despite a small sample size we observed changes in neuromarkers and in hepatic stiffness in patients randomized to the experimental arm. We observed changes in CSF biomarkers (lower phosphorylated-tau and higher neopterin and NFL) that need to be replicated in large cohorts. Subclinical neurotoxicity may be observed in patients with HAND and warrants prospective studies.

**Graphical Abstract:**

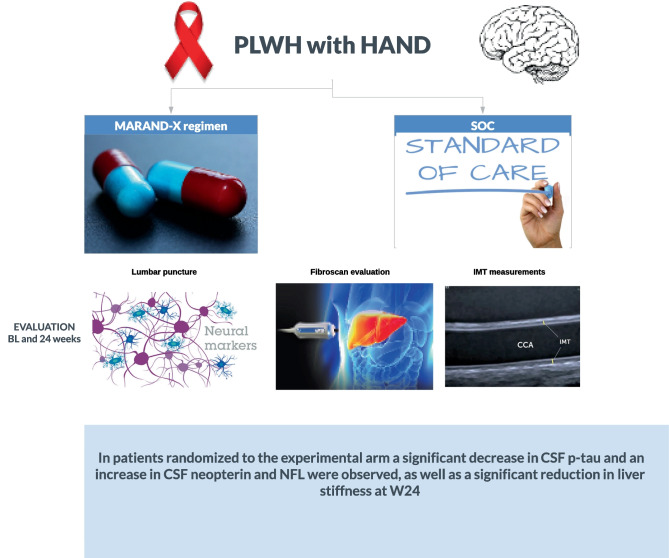

**Supplementary Information:**

The online version contains supplementary material available at 10.1007/s11481-023-10086-7.

## Introduction

Since the introduction and widespread use of highly active antiretroviral therapy [HAART], incidence and prevalence of central nervous system [CNS] opportunistic infections and HIV-associated dementia [HAD] significantly diminished (Nightingale et al. 2014). Nevertheless, successfully treated people living with HIV [PLWH] often suffer from neurocognitive impairment [NCI] whose prevalence varies according to age, geographical setting, and diagnostic criteria (Nightingale et al. 2014). According to recent data from multiple European cohorts, the prevalence of HIV-associated neurocognitive disorders [HAND] varies between 27% and 49.8%, with the vast majority of cases being represented by asymptomatic neurocognitive impairment [ANI] (Metral et al. 2020; Haddow et al. 2018; Trunfio et al. 2018; Cysique et al. 2021; Vassallo et al. 2017). HAND is a multifactorial disease linked both to HIV and host-related factors, and its precise pathogenesis is still incompletely understood. Recent studies also suggest that vascular pathology, especially highly prevalent small vessels diseases, may influence the development and severity of neurocognitive disorders (Cysique et al. 2021). Additionally, patient- (genetics, coinfections, education and cognitive reserve among others), HIV- (low nadir CD4 + lymphocyte count, high HIV-DNA, incomplete compartmental virological control and persistent inflammation) and treatment-associated factors (antiretrovirals neuropenetration/neuroefficacy and potential neurotoxicity) have been shown to interact with neurological performance of PLWH. (Antinori et al. 2007; Calcagno et al 2018; Ciccarelli et al. 2011; Underwood et al. 2015; De Benedetto et al. 2020; Alford et al. 2021). Despite the ongoing debate on HAND diagnostic criteria, there is preliminary evidence that even milder forms might negatively affect patients’ quality of life, treatment adherence and long-term outcomes (Cysique et al. 2021; Alford et al. 2021). Diagnosing HAND necessitates a thorough evaluation conducted by a skilled neuropsychologist, which can be time-consuming and susceptible to various confounding factors, and may also be impacted by the learning effect (Cysique et al. 2021). In recent years efforts have been made to identify more objective diagnostic criteria of HAND that include serum and cerebrospinal fluid (CSF) biomarkers and instrumental tests. A single marker that effectively differentiates PLWH with or without HAND has not yet been identified despite promising results have been attained with certain neuronal damage and inflammation biomarkers (Bandera et al. 2019; Motta et al. 2017). On the contrary, carotid artery intima-media thickness (IMT) and fibroscan are important and validated tools to non-invasively measure atherosclerotic features, liver stiffness and fat content (Msoka et al. 2019; Lemoine et al. 2019; Valcour et al. 2016; Ciccarelli et al. 2014; Han et al. 2013). HIV infection per se as well as concomitant infections and the associated chronic immune activation indeed increase cardiovascular risk (Msoka et al. 2019); this in turn can affect the endothelial (including blood–brain barrier) and liver functions (including the metabolism of neurotoxic substances) thereby contributing to neurocognitive impairment (Msoka et al. 2019; Lemoine et al. 2019; Valcour et al. 2016; Ciccarelli et al. 2014; Han et al. 2013). The idea of utilizing non-invasive tools to indirectly assess neurocognitive decline is intriguing. However, there have been limited efforts so far to combine screening tools for cardiovascular risk and liver functioning with data that directly relates to intrathecal phenomena, such as CSF biomarkers.

Aim of this study was to describe whether and how CSF biomarkers of astrocytosis, immune activation and neurodegeneration, IMT and Fibroscan values change in patients randomized to a less-neurotoxic antiretroviral treatment (darunavir/cobicistat plus emtricitabine plus maraviroc) and to assess potential inter-relationships between the dynamic changes eventually observed. Not being a standard one, the choice of this regimen has been based on relevant data. In particular, in an in vitro model on rat neurons, all antiretroviral drugs were associated with a certain degree of functional damage, although those with a milder effect were found to be emtricitabine, tenofovir, darunavir, and maraviroc (Robertson et al. 2012). These neurotoxic effects may become apparent once the inflammatory effect resulting from viral replication subsides, typically after months or years from the start of ART. Maraviroc is a CCR5 receptor inhibitor approved for the treatment of HIV infection (in combination with other drugs) and possesses interesting anti-inflammatory, immunomodulatory, and beneficial effects on small blood vessels. Some evidence has shown an improvement in neurocognitive function, certain markers of inflammation in cerebrospinal fluid, and spectroscopic magnetic resonance values in HIV-positive patients who were given maraviroc in addition to their treatment (Kelly et al. 2013; Garvey et al. 2012; Vera et al. 2012; Ndhlovu et al. 2014). A small randomized and controlled trial confirmed these results: in 9 patients whose therapy was intensified with maraviroc, a significant improvement in neurocognitive function was observed (d = 0.55, 95%CI -0.47–1.55) (Gates et al. 2016).

## Materials & Methods

This manuscript reports the baseline and week 24 secondary analysis and outcomes of a randomized controlled clinical trial, MARAND-X, clinical trial number NCT03163277 registered on www.clinicaltrial.gov, 19/05/2017 (Barco et al. 2022). Patients signed a written informed consent before being enrolled and the study was approved by the Ethics Committee of the “Città della Salute e della Scienza, Turin, Italy, 179/2016/U. Briefly, participants were double blind block 1:1 randomized to either switching to a less neuro-toxic regimen (based on in vitro evidence and consisting of darunavir/cobicistat plus emtricitabine plus maraviroc; intervention arm, based on data from Kelly et al. 2013; Garvey et al. 2012; Vera et al. 2012; Ndhlovu et al. 2014; Gates et al. 2016, as described in the introduction section) or to maintaining their current regimen (standard of care, SOC arm). Inclusion criteria were: age ≥ 18 years, diagnosis of HAND according to Frascati’s criteria (REF), having been on HAART for a minimum of 6 months, showing no signs of significant resistance-associated mutations in both plasma and cerebrospinal fluid (CSF), and with plasma and CSF HIV-RNA < 50 copies/mL. Exclusion criteria were: being on regimens containing efavirenz and/or darunavir, having a CXCR4- or dual/mixed-tropic virus (or an indeterminate tropism) on genotypic or phenotypic test before the start of a continuously effective HAART, or on a genotypic test performed on proviral DNA in the previous 6 months; the presence of any condition potentially able to affect the neurocognitive performance (intellectual disability, brain trauma, cerebrovascular events, liver cirrhosis, renal failure with estimated creatinine clearance < 50 ml/min, current or past systemic or CNS infectious, autoimmune, neurodegenerative, or neoplastic diseases; the presence of untreated severe anxiety-depressive syndrome; alcohol or narcotic/psychotropic substance abuse in the previous three months); limited Italian language skills or any inability to undergo neurocognitive tests, pregnancy and breastfeeding, clinical need of drugs prohibited in co-administration with the antiretrovirals used in the study. The demographic and clinical characteristics of these patients were recorded and monitored from baseline (BL) to week 24 (W24). Derived variables (such as AST to Platelet Ratio Index score, APRI, and Fibrosis-4, FIB-4) were calculated accordingly. APRI was calculated through [(AST/upper limit of the normal AST range) X 100]/Platelet Count formula; FIB4 trough Age (years) × AST (U/L)/[platelets(10^9^/L) × ALT^1/2^(U/L)] formula. Further details on the study protocol, population and methods can be found in Barco et al. 2022.

### IMT and FIBROSCAN

Patients were evaluated by the same ultrasonographist for both IMT and Fibroscan at baseline and then at W24 after randomization. Fibroscan is a quick, non-invasive technique used to measure liver stiffness (kPa), which correlates with fibrosis. To achieve a valid liver stiffness evaluation (LSE) the operator obtained all the following three criteria: (1) ≥ 10 successful liver stiffness measurements; (2) IQR/median ratio < 0.30 and (3) ≥ 60% measurement success rate through FibroScan ® 502 Touch (Vibration-Controlled Transient Elastography (VCTE™), Echosens). The carotid intima-media thickness (IMT) is a widely used surrogate marker for atherosclerosis worldwide, and can be simply, noninvasively, and reproducibly measured through B-mode carotid ultrasound. A high-resolution B-mode system, equipped with a linear array transducer > 7 MHz with minimal compression (< 10:1) and footprint of at least 3 cm (Esaote MyLab™ 25 Gold) was applied to measure IMT in triplicate, sampling at ± 1 cm from carotid bulb bifurcation. The average value of 3 intima-media measures (in mm) was retained.

### Cerebrospinal Fluid Analysis

Atraumatic needles were used for lumbar punctures (LPs). CSF was analyzed for cell count and several biomarkers. CSF total tau (t-tau), phosphorylated tau (p-tau), and β-amyloid 1–42 fragment (Aβ_1-42_) were measured by immunoenzymatic methods (Innotest, Fujirebio Europe, Belgium) with limits of detection (LoD), respectively, of 34, 15.6, and 65 pg/ml. Neopterin and S100-beta were measured through validated ELISA methods (DRG Diagnostics, Marnurg, Germany, and DIAMETRA Srl, Spello, Italy, respectively). Serum and CSF Neurofilament light chain (NFL) were evaluated with a new digital immunoassay, the Single Molecule Array technology (Simoa SR-X, Quanterix®, Billerica, USA) with the NF-light advantage® assay (LoD 0.038 pg/mL). Reference values were as follows: t-tau < 300 pg/mL (in patients aged 21–50), < 450 pg/mL (in patients aged 51–70), or < 500 pg/mL in older patients, p-tau (< 61 pg/mL), Aβ_1-42_ (> 500 pg/mL), neopterin (< 1.5 ng/mL) and S100-beta (< 380 pg/mL). CSF indexes of blood–brain barrier permeability (CSF to serum albumin ratio, or CSAR) were deemed normal when < 6.5 in patients aged < 40 or < 8 in patients > 40 years (20). HIV-RNA was quantified by the Roche Amplicor assay v2.0 (Hoffman-La Roche, Basel, Switzerland) with a lower limit of quantification of 20 copies/ml.

### Statistical Analysis

Pseudo-anonymazed data were collected in an encrypted database; statistical analyses were performed using SPSS statistic (version 27.0 IBM Corp.). Data are described as number (percentage) or median (interquartile range, IQR). Non-parametric tests were used for the analysis according to categorical or continuous nature of the variables (Chi-square test, Fisher’s exact test, Spearman’s rank correlation, and Mann–Whitney test). The intra-subject median change from baseline to W24 was analyzed by paired Wilcoxon test; significance for changes in binary and multinomial variables were analyzed by the symmetry McNemar chi-square test and by the marginal homogeneity test, respectively. The Delta of the variables of interest was calculated as the difference between week 24 and baseline median values. The study was designed as an analysis of a continuous variable between two independent groups of subjects (experimental arm and control arm). The reference study is the one mentioned earlier by Gates and colleagues (Gates et al. 2016), which reported an effect size of 0.55 with a normal distribution (though with high variability and wide confidence intervals). Based on this data and using a conservative estimate, a difference of 0.15 between the arms is hypothesized, with a standard deviation of 0.2. Therefore, it was estimated that 38 subjects per arm needed to be studied to be able to reject the null hypothesis with a power of 90%. The probability of a Type I error associated with this test is set at 5%.

## Results

### Study Population

Participants were enrolled between August 2017 and December 2019; the last follow-up visit was in June 2020, when the study was prematurely terminated because of slow accrual worsened by COVID-19 pandemic. Of 53 enrolled participants, 28 completed the follow-up and were included in the longitudinal analysis, as shown in Table 1.

**Table 1:** Demographic and clinical characteristics Baseline and W24, by arm allocation

	Overall n = 28	MARAND-X n = 15	SOC n = 13	p-value
**Demographics**				
Age (years)	57 (53 – 60)	57 (53 – 61)	55 (53 –59)	0.729
Gender (male)	21 (75)	10 (66.7)	11 (84.6)	0.39
Ethnicity				1
African	1 (3.6)	1 (6.7)	0 (0)	
Caucasian	25 (89.3)	13 (86.7)	12 (92.3)	
Latin	2 (7.1)	1 (6.7)	1 (7.7)	
Education	10 (8 – 13)	8 (7 – 12)	13 (10 – 13)	0.159
BMI	24 (23 – 27)	24 (22 –25)	25 (24 – 29)	0.084
**Comorbidities**				
Hypertension	6 (21.4)	3 (20)	3 (23)	1
Type 2 Diabetes	1 (3.6)	1 (6.7)	0 (0)	1
Smoking	7 (25)	4 (26.7)	3 (23.1)	1
Depressive disorder	6 (21.4)	5 (33.3)	1 (7.7)	0.173
HBV co-infection	4 (14.3)	1 (6.7)	3 (23.1)	0.311
HCV former infection	7 (25)	4 (26.7)	3 (23.1)	1
eGFR-EPI-CKD (ml/min/1.73m^2^)	92 (78 – 100)	91 (81 – 100)	95 (75 – 100)	0.964
**HIV-1 infection**				
Time from HIV-1 diagnosis (years)	16 (8 – 25)	14 (7.5 – 24)	16 (8 – 25)	0.344
VL < 50 cp/mL				
Plasma	28 (100)	15 (100)	13 (100)	1
CSF	28 (100)	15 (100)	13 (100)	1
Time with VL < 50 cp/mL (years)	11 (5.5 – 15)	10 (5 – 13)	13 (6.8 – 16)	0.352
CD4^+^ T cells				
Cells count nadir (cells/μL)	300 (150 – 420)	320 (150 –400)	250 (180 –440)	0.89
Current cell count (cells/μL)	710 (480 – 890)	560 (450 – 800)	740 (540 – 1000)	0.254
Current Percentages (%)	32 (24 – 42)	31 (24 – 40)	36 (28 – 42)	0.549
Current CD4/CD8 ratio	0.8 (0.65 – 1.5)	0.8 (0.5 – 1.3)	0.8 (0.7 – 1.6)	0.562
**Pre-screening ARV regimen**				0.109
INSTI + 2 NRTI	15 (57.1)	10 (66.7)	5 (38.5)	
NNRTI + 2 NRTI	9 (28.6)	3 (20)	6 (46.2)	
PI + 2 NRTI	2 (7.1)	0 (0)	2 (15.4)	
INSTI + PI	2 (7.1)	2 (13.3)	0 (0)	
CPE	6 (6 – 8)	6 (6 – 8)	6 (6 – 8)	1
**Neurocognitive disorder**				
HAND				0.6
ANI	24 (85.7)	12 (80)	12 (92.3)	
MND	4 (14.3)	3 (20)	1 (7.7)	
HAD	0 (0)	0 (0)	0 (0)	
GPS	2.5 (1.5 – 3.1)	2.3 (1.7 – 3.1)	2.6 (1.5 – 3.0)	1

Data are presented as median (interquartile ranges) or number (percentage). *ANI* Asymptomatic neurocognitive impairment, *ARV* antiretroviral, *BMI* body mass index, *CD* cluster ofdifferentiation, *CPE* CNS penetration-effectivness score, *eGFR* estimated glomerular filtrate rate, *GPS* Global Performance Score, *HAD* HIV associated dementia, *HAND* HIV associated neurocognitive disorder, *INSTI* Integrase strand transfer inhibitors, *MARAND-X* Interventional arm, *MND* Mild neurocognitive disorder, *NNRTI* non-nucleosidic reverse transcriptase inhibitors, *NRTI* nucleosidic reverse transcriptase inhibitors, *p* p-value, *PI* protease inhibitors, *SOC* Standard of care (control arm), *VL* HIV-1 viral load

At baseline, the two arms did not differ for any demographic nor viro-immunological parameter or for HAART regimens: SOC were more commonly on NNRTIs, whereas MARAND-x arm had a larger proportion of participants coming from INSTI-based regimens, with no statistically significant difference (Table 1). The complete breakdown of antiretrovirals is available.

### Longitudinal Analysis

No relevant changes in viro-immunological parameters were observed between baseline and W24 either in the overall population and in both the study arms. We observed only a mild decrease in CD4/CD8 ratio in the SOC (from baseline 1.1 to 0.8 at W24; Supplementary Table 2). All the study participants maintained plasma and CSF suppression at W24, and during the study period. 

### Neuromarkers

**Fig. 1:**
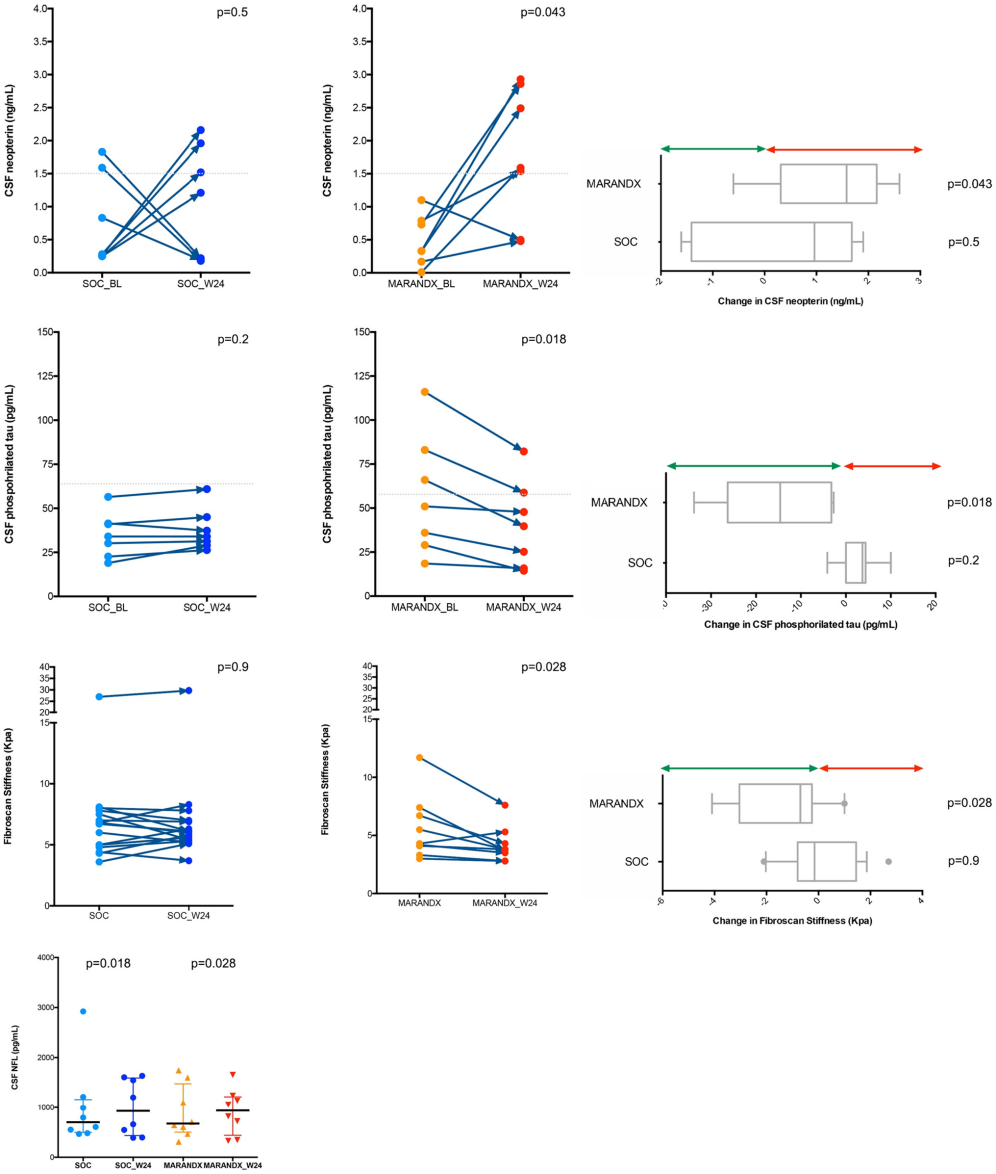
Neopterin, CSF neopterin, pTau, liver stiffness and NFL variation during study period, and between groups, at BL and W24. Arms: 0: SOC; 1: MARAND-X

In the whole study population, we observed significant changes over time in CSF neopterin and NFL (p = 0.048 and p = 0,001, respectively; Supplementary Table 2 and Fig. 1). When stratifying for study arm, we observed a significant increase in CSF neopterin and decrease in ptau in the intervention arm (p = 0,043 and p = 0.018), whereas CSF NFL increased in both (p = 0,018 SOC; p = 0,028 intervention; Supplementary Table 2 and Fig. 1). No other CSF biomarkers significantly varied over time, nor between groups.

### IMT and Lipidic Profile

No significant differences were observed over time and between groups in terms of IMT and lipidic profile.

### Fibroscan, Liver Biomarkers and Scores

A reduction in the APRI score, AST, and stiffness were observed in the MARAND-X arm and not in the SOC arm, as shown in Supplementary Table 2. On the contrary, in the SOC arm, there was a statistically significant increase in ALT, although it was not clinically relevant. The other liver function tests remained unchanged compared to the baseline. Additionally, Delta stiffness showed a trend towards change (p = 0.02), while Delta CAP did not show such a trend (Supplementary Table 3).

### Correlations Among Biomarkers and Changes Over Time

**Fig. 2:**
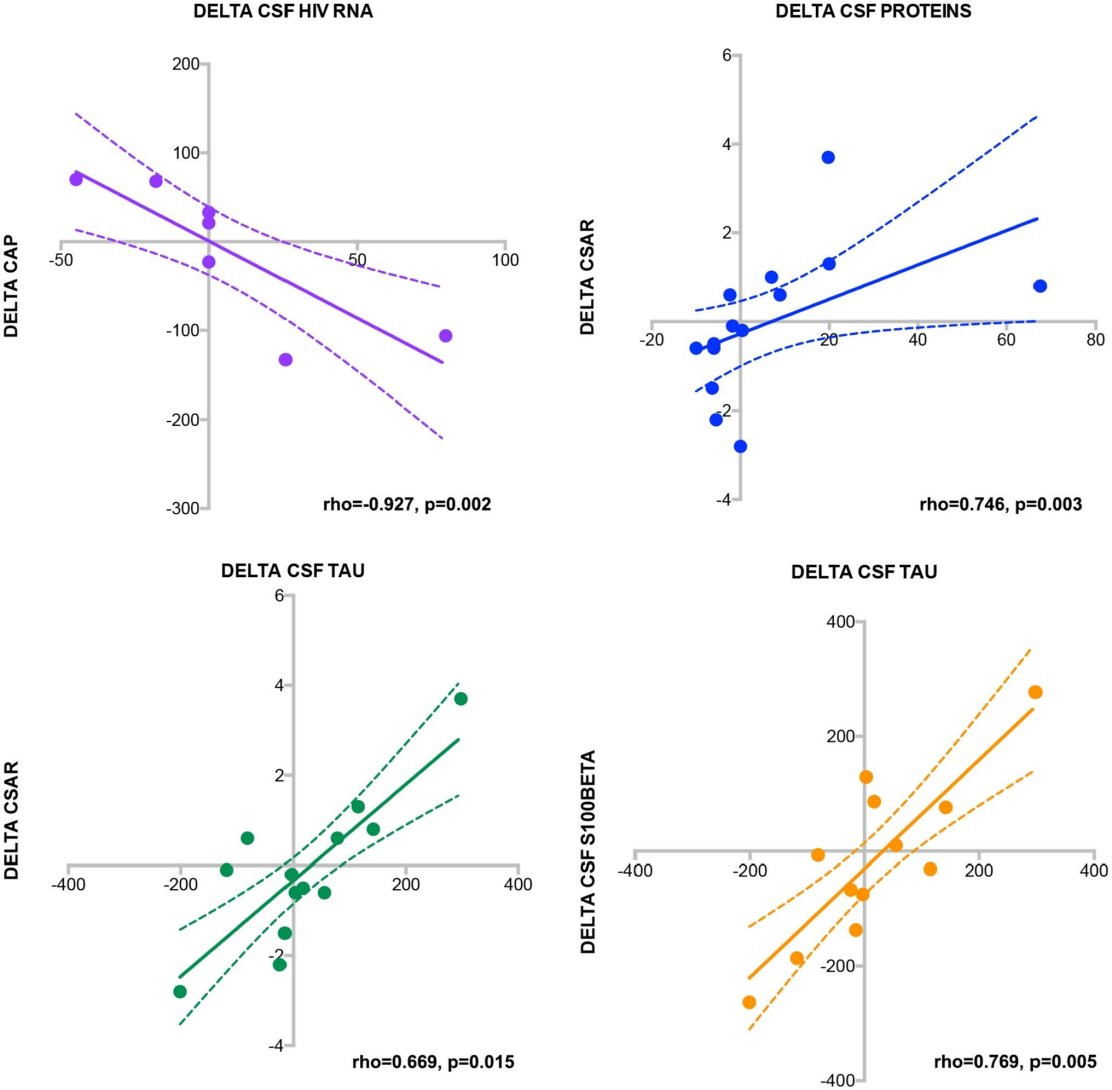
Correlations among biomarkers and changes over time. CSF total tau correlated with CSF s100Beta (rho = 0.769, p = 0.005) and with changes in CSAR (rho = 0.669, p = 0.015)**,** while CSF protein changes were associated with CSAR modifications (rho = 0.746, p = 0.003), and changes in CAP values correlated with CSF HIV-RNA (rho = -0.927, p = 0.002)

Lastly, we assessed whether delta changes over time among the variables of interest were correlated with each others: selected statistically significant association are depicted in Fig. 2. We observed significant association between changes in CSF total tau and in CSF s100Beta (rho = 0.769, p = 0.005) and with changes in CSAR (rho = 0.669, p = 0.015)**.** As expected, CSF protein changes were associated with CSAR modifications (rho = 0.746, p = 0.003). We then explored the potential association between changes in delta liver stiffness (kPa) and delta steatosis (CAP) with changes in CSF biomarkers (besides being associated between them with rho = 0.833, p = 0.005). The only statistically significant association we observed was between changes in CAP values with CSF HIV-RNA (rho = -0.927, p = 0.002) modifications over time.

## Discussion

We observed that six months after switching to a less neurotoxic regimen, participants in the intervention arm had higher CSF levels of neopterin and NFL, no changes in IMT and lipid profile, and a modest improvement in liver function tests and fibrosis scores (in comparison to participants remaining on previous regimen). Our hypothesis was that switching towards drugs that had a lower in vitro neurotoxicity would be associated with better cognitive function in patients with HAND as well as an improvement in biomarkers of neuronal damage. Yet robust evidence of an in vivo neuronal protection is lacking as well as the effect on lipid profile, cardiovascular risk and liver function tests given the use of boosted darunavir, maraviroc and emtricitabine (a triple combination seldom used in clinical practice as well as in medical literature). While the results of the primary objective (neurocognition) have been submitted for publication the other endpoints were mostly exploratory and the sample size was, unfortunately, very small.

HAND is a complex phenotype and the path towards effective treatments relies on the identification of pathogenetic mechanisms and reliable biomarkers. We included IMT, lipid, liver scores, and CSF markers in order to understand the potential underlying mechanisms (and response to treatment change) of vascular, hepatic and neurological factors in the neurocognitive impairment observed in PLWH (Msoka et al. 2019; Lemoine et al. 2019; Valcour et al. 2016; Ciccarelli et al. 2014; Han et al. 2013). The majority of the enrolled subjects were diagnosed with ANI, while only a minority showed MND and none presented HAD, similarly to what is currently globally observed (Haddow et al. 2018; Calcagno et al. 2018). Despite this, almost one fourth of the subjects did not complain about neuropsychological symptoms at the screening visit. This finding confirms previous studies, which observed a lack of disease awareness and insight resulting in a discordance between objective tests results and patients’ perceptions and the need of more reliable diagnostic tools (Cysique et al. 2021). In our population we did not observe significant virological changes over time.

CSF biomarkers that reflect processes within the brain constitute an important complementary approach to evaluate ongoing CNS disease. In our study, considering the similar cognitive profiles between HAND and neurodegenerative dementias, Tau, pTau, s100b, beta40, beta42, NFL and neopterin were evaluated, as well as CSF proteins, glucose and cells.

From the literature, we know that neopterin is an early marker of intrathecal immune response, whose CSF levels are elevated in most HIV subjects compared to uninfected individuals: recent studies showed an elevated neopterin levels in patients with HAND (Edén et al. 2016). In our study we observed a statistically significant increase in CSF neopterin in the intervention arm, possibly not confirming the observation that a less neurotoxic regimen may lead to less immune-activation and that other factors that we could not identify may be additionally involved, independently. Importantly, even if rising, neopterin stayed below the threshold value that is considered normal in both groups for the majority of patients despite a few outliers. Data from literature have proven the importance of a low-level immune-activation in driving alterations in functional brain activities, possibly expressed by the finding of a raised neopterin in the CSF, but no definitive data exist for beneficial lower levels of this protein in HAND patients (Edén et al. 2016). Other hypothesis to explain that finding may be ascribable to the chosen drug regimen: eliminating an NRTI from the HAART regimen may be deleterious in patients with HAND, since it is hypothesized that neurotoxicity associated with antiretroviral drugs could be class-specific and NRTIs have been shown to block inflammasomes and lower the likelihood of β-amyloid plaque deposition in the frontal cortex thus raising the possibility of a protective effect of NRTIs on the development of neurodegeneration (De Benedetto et al. 2020); still, as assessed in prospective studies, patients in dual therapy don’t seem to underperform in this specific group (Pérez-Valero et al. 2018; Mondi et al. 2015; Trunfio et al. 2020). An alternative hypothesis is that the intervention regimen has a less potent effects on controlling neuroinflammation. Ultimately, another compatible explanation lays on a possible lower adherence due to the increased complexity of the regimen; unfortunately, we could not register directly any of these variables for our patients (dedicated questionnaires), but indirect tools (viral load) to measure adherence confirmed an overall excellent compliance (all patients remained virologically suppressed). We also observed changes in CD4/CD8 ratio since this biomarkers has been previously associated with HIV-associated non infectious comorbidities and HAND (Vassallo et al. 2013, 2017): yet no significant changes over time was recorded.

NFL is a neuronal cytoplasmic protein highly expressed in large calibre myelinated axons. One of its main roles is to maintain axonal caliber and facilitate effective nerve conduction; its level increase in CSF and plasma proportionally to the degree of axonal damage in many neurological disorders, including inflammatory, neurodegenerative, traumatic and cerebrovascular (Mielke et al. 2019). Elevated NFL is one of the markers with a more consistent correlation with NCI in HIV (Anderson et al. 2018; Guha et al. 2019; Gisslén et al. 2015). Similarly to what we observed with neopterin, we noticed a slight elevation in both groups during the study period. In our case, that may partially be explained with the fact that patients were treated with old drug combinations, which would now be considered suboptimal, or because, as already discussed, omitting NRTIs in HAND patients or complicating a drug regimen with more pills may lead to worse results and lower adherence. The link between HAND and poor adherence has been recently confirmed by a systemic review and meta-analysis (Nweke et al. 2022). In addition, another possible explanation we could not assess pertains to the viral CNS reservoir of these patients that may drive such alterations independently of treatment change: indeed intensification studies failed in demonstrating a clear beneficial effect. (Sacktor et al. 2018; Yilmaz et al. 2010). No plausible explanations for this finding can be further speculated based upon our data. Still, in the absence of unequivocal data for the NFL trajectory in HAND, these two markers may rise independently from the means applied here in this randomized clinical trial and a single intervention may not be sufficient in patients that already suffer from a low-level ongoing neuronal damage, frequently started years prior to any active medical intervention. A potential bias for having an elevated NFL in the absence of actual HIV-replicative-dependent harm may be the LP itself: in macaque monkeys an increase the NFL value by 162% was registered after LPs; that subsequently returns to normal in 1 to 2 months from the spinal tap. These data are not confirmed up-to-date in humans nor in PLWH (Boehnke et al. 2020).

Tau protein is abundant in the central nervous system and involved in microtubule assembly and stabilization. It is predominantly associated with axonal microtubules and present at lower level in dendrites where it is engaged in signaling functions. Tau it is generally phosphorylated (ptau) during its physiological functioning. Generally speaking, increased phosphorylation results in decreased binding to microtubules which is important in tau-mediated neurodegeneration. Tau protein is also involved in blood–brain barrier dysfunction that was described in numerous neurological conditions and possibly play such role in infectious pathologies of the CNS (Mietelska-Porowska et al. 2014; Calcagno et al. 2016). The finding of this protein in the CSF it is ascribable to neuronal damage generally, and particularly in what happen in dementia-like CNS pathological entities. In the setting of HAND the link with AD (characterized by amyloid deposition and tau pathology) has been repeatedly suggested, still maintaining consistent differences (Trunfio et al. 2022). On the opposite from what we observed with neopterin and NFL, ptau decreased only in the intervention arm. In view of all that factors, the finding of a lower ptau in patients undergoing a less potentially neurotoxic regimen is of great relevance given the significantly higher risk of dementia observed in elderly PLWH (Bobrow et al. 2020; Lam et al. 2021; Yu et al. 2023). especially because baseline ptau resulted higher in this same patients’ group and this protein suffer less influence from macrophage activation. A reduced level during the switch regimen may be not sufficiently significant, as we did not observe the same improvement for what pertains to total tau and beta-amyloid.

Besides cellular damage and pathway alterations we explored vascular and liver involvement and changes with treatment modifications. When it comes to peripheral markers and non-invasive tool, we observed that IMT did not vary significantly over time. This may be of no surprise, as typically IMT variation may take more time and present alterations later than the 24 weeks study period herein considered. Yet the use of maraviroc was potentially beneficial. It had been associated with significant reduction in IMT, pulse wave velocity and triglycerides, and to have additional cardiovascular and neuroprotective characteristics that may have played a role in this study but were not readily measurable (Piconi et al. 2016; Martin-Blondel et al. 2016; Francisci et al. 2019).

Finally we explored whether changes in treatment regimen could be beneficial for liver involvement as measured by non-invasive biomarker of liver function. The hypothesis was a link between liver and brain function, involving the gut-brain axis and macrophage functions in systemic inflammation. (Thomas and Apovain 2017; Spencer et al. 2010). In fact, formerly maraviroc has been associated with benefit over fibrosis progression, specifically for prolonged treatment (Gonzales et al. 2014). As previously mentioned, additional HIV-related risk factors for HAND include immune-activation, dysregulation of the gut microbiome, and the potential toxicity of ART. On the other hand, non-alcoholic fatty liver disease (NAFLD) has become increasingly significant in PLWH. Thus, there is growing recognition of the need to address underlying lifestyle factors, such as weight reduction and optimal glycaemic control, along with pharmacological interventions. As part of the expanding interest in novel approaches, maraviroc has been proposed. Maraviroc works by inhibiting the binding of HIV-1 gp120 to the CCR5 coreceptor, thereby preventing the virus from entering cells. Its ability to antagonize CCL5-CCR5-mediated interactions has sparked interest in its potential anti-inflammatory benefits, in addition to its anti-HIV activity, particularly in the liver and brain (Martin-Blondel et al. 2016; Bradshaw et al. 2020; Gonzales et al. 2014).

Notably, the chemokine CCL5/RANTES, which acts as the ligand for CCR5, plays a crucial role in hepatic inflammation and fibrosis. CCR5 facilitates interactions among intrahepatic immune cells, promoting activation and migration of Kupffer cells and hepatic stellate cells, which, in turn, trigger inflammation and hepatic fibrosis. Consequently, inhibiting this pathway could potentially reduce the progression of fibrosis (Bradshaw et al. 2020). This is relevant in view of the fact that liver fibrosis is a process damaging lymphnodes, lymphatic function and immune-regulation, and there are similarities between the intestinal and the blood–brain barrier. Since the measurement of these markers is notably convenient and non-invasive, our observation of a reduction in liver stiffness becomes particularly significant within the context of the aforementioned hypothesis. It ultimately further supports the idea of a potential role for maraviroc therapy and demonstrates that readily accessible tools can be employed to comprehensively assess PLWH with HAND.

Putting these observations together, we should highlight the importance of following-up patients comprehensively with easy-to-use, informative and reproducible non-invasive tools that may provide important information on the patient’s status.

Several limitations should be recognized. It should be emphasised that the number of patients in both intervention and control arm is small, hence more data may be required from bigger study population observed over longer time to detect additional differences. Population was also heterogenous, as many of the enrolled subjects had comorbidities, in particular some suffered from depressive disorders (Hammond et al. 2016) and hypertension, or others being active smokers; that could partially act as confounding factors (specially on IMT), which has been already widely associated to neurocognitive decline (Calcagno et al. 2018). Moreover, a minority of patients enrolled were previously HCV-positive, which is a potential bias for liver and lipid scores calculation, a potential driver of immune-activation, and may impact on stiffness results; despite that, they all had been treated long before the enrollment and no one showed liver cirrhosis. It is still unclear if HCV eradication also eliminate the burden of disease on cognition, or if it has permanent consequences on CNS (Underwood et al. 2015). In addition, the lack of well-defined markers of immune activation and general inflammation is generally recognized. These processes can persist in patients on ART, and both have been implicated in neurocognitive impairment and HAND in general. Inclusion of more inflammatory markers such as IP-10, sCD163, CD38/DR on CD8 T cells would have aided in interpretation. Unfortunately, we could not assess these in our cohort.

However, this study also has strengths: our cohort had demographic characteristics similar to other modern study groups and our results could therefore be extended to minimally confounded PLWH diagnosed with HAND. It is the first real life project on HAND coupling a non-invasive and invasive tools for the evaluation of neurocognitive field, with other classical non-invasive tool such as IMT and Fibroscan that have been largely validated across cohorts (Msoka et al. 2019; Lemoine et al. 2019; Valcour et al. 2016; Ciccarelli et al. 2014; Han et al. 2013). More data from the principal analysis are possibly going to elucidate the relationship between these tools and neurocognitive performances.

## Conclusions

In conclusion we observed minor changes in CSF and hepatic biomarkers and no change in IMT after 24 weeks of switch to a less neurotoxic regimen that included emtricitabine, darunavir/cobicistat and maraviroc. Some changes were potentially deleterious, as we observed rising in neopterin and NFL, while others appear promising, since we registered lower ptau, stiffness and APRI values in the intervention arm. We could not observe a strict relationship between CNS and liver function. A more extensive investigation regarding the gut-brain axis and other markers analyzing the role of liver stiffness, inflammatory and neuromarkers should be implemented in larger cohorts to better characterize this plausible still unproven hypothesis.

### Supplementary Information

Below is the link to the electronic supplementary material.

Supplementary file1 (DOCX 43 KB)

## Data Availability

The data that support the findings of this study are not openly available due to reasons of sensitivity and are available from the corresponding author upon reasonable request. Data are located in controlled access data storage at “Città della Salute e della Scienza, Turin, Italy, 179/2016/U, and are available at clinical trial number NCT03163277 registered on www.clinicaltrial.gov, 19/05/2017.

## Abbreviations

HAND: HIV associated neurocognitive disorder
PLWH: people living with HIV
APRI AST: to platelets ratio index
IMT: intima-media thickness
CSF: cerebrospinal fluid
NFL: neurofilament
CNS: central nervous system
