# Supplementary material for: Changes in Cerebrospinal Fluid, Liver and Intima-media-thickness Biomarkers in Patients with HIV-associated Neurocognitive Disorders Randomized to a Less Neurotoxic Treatment Regimen
Source: J Neuroimmune Pharmacol. 2023 Oct 31;18(4):551–62. doi: 10.1007/s11481-023-10086-7 (PMC10770227; doi:10.1007/s11481-023-10086-7)
Supplement: Supplementary file 1 — Supplementary file1 (DOCX 43 KB) [file 11481_2023_10086_MOESM1_ESM.docx]

| **Patient number** | **Pre-enrollment therapeutic regimen** | **Allocation Arm** |
| --- | --- | --- |
| **1** | TAF/FTC+NVP | SOC |
| **2** | TDF/FTC/RPV | MARAND |
| **3** | ABC/3TC/DTG | SOC |
| **4** | ABC/3TC/DTG | SOC |
| **5** | TAF/FTC/EVG/c | MARAND |
| **6** | TAF/FTC+DTG | MARAND |
| **7** | TAF/FTC/EVG/c | MARAND |
| **8** | TAF/FTC/EVG/c | MARAND |
| **9** | TAF/FTC/DRV/c | SOC |
| **10** | TAF/FTC/RPV | SOC |
| **11** | DRV/c+RAL | MARAND |
| **12** | ABC/3TC/DTG | MARAND |
| **13** | TAF/FTC/EVG/c | SOC |
| **14** | DRV/c+RAL | MARAND |
| **15** | DTG/ABC/3TC | MARAND |
| **16** | TAF/FTC+DTG | SOC |
| **17** | TAF/FTC+ATV/r | SOC |
| **18** | TAF/FTC/RPV | SOC |
| **19** | TAF/FTC/RPV | MARAND |
| **20** | TAF/FTC + DTG | MARAND |
| **21** | TAF/FTC + DTG | MARAND |
| **22** | TAF/FTC/RPV | SOC |
| **23** | TAF/FTC+RAL | MARAND |
| **24** | TAF/FTC+DTG | SOC |
| **25** | TAF/FTC/RPV | MARAND |
| **26** | TAF/FTC/RPV | SOC |
| **27** | TAF/FTC + NVP | SOC |
| **28** | ABC/3TC/DTG | MARAND |

**Supplementary** **table 1** Baseline regimens, complete breakdown of antiretrovirals of all participants. SOC: standard of care; ABC: abacavir; ATV/r: atazanavir/ritonavir; c:cobicistat; DRV: darunavir; DTG: dolutegravir; EVG: elvitegravir; FTC: emtricitabine; NVP: nevirapine; RPV: rilpivirine; TDF: tenofovir disoproxil fumarate; TAF tenofovir alafenamide; 3TC: lamivudine;

|  | **BL Overall (n=28)** | **W24 Overall** | **p=** | **BL SOC**  **(n=13)** | **W24 SOC** | **p=** | **BL MARAND-X**  **(n=15)** | **W24 MARAND-X** | **p=** |
| --- | --- | --- | --- | --- | --- | --- | --- | --- | --- |
| **IMT (mm)** | 0.66 [0.87;0.55] | 0.7 [0.78;0.54] | 0.6 | 0.65 [0.97;0.5] | 0.59 [0.93;0.56] | 0.7 | 0.66  [0.84;0.6] | 0.64  [0.72;0.5] | 0.7 |
| **Stiffness (kPa)**  **CAP (dB/m)** | 5.7 [6.9;4.3]  258 [299;195] | 5.5 [6.45;3.8]  250 [293;217] | 0.1  0.5 | 6.8 [7.5;4.6]  255 [296;200] | 6.6 [5.97;5.32]  244 [303;222] | 0.9  0.8 | 4.9  [6.3;4.12]  251  [313;192] | 3.8  [5.3;3.2]  246  [274;176] | **0.028**  0.3 |
| **FIB4** | 1.19 [1.0;1.6] | 1.14 [0.94;1.28] | 0.2 | 1.21 [1.03;1.62] | 1.12 [0.93;1.26] | 0.5 | 1.19 [0.95;1.98] | 1.14 [0.89;1.31] | 0.8 |
| **APRI** | 0.23 [0.19;0.32] | 0.22 [0.17;0.3] | 0.3 | 0.23 [0.2;0.33] | 0.27 [0.22;0.34] | 0.6 | 0.23 [0.17;0.31] | 0.18 [0.14;0.24] | **0.014** |
| **TG (mg/dl)** | 123 [98,75;167,5] | 131 [100,25;179,25] | 0.5 | 119 [84.5;161.5] | 131 [97;179] | 0.1 | 135 [107.5;175.75] | 132 [103;179.5] | 0.5 |
| **Col tot**  **(mg/dl)** | 191,5 [175,5;214,75] | 188 [168,75;214,25] | 0.6 | 184 [168.5;206.5] | 188 167;212][ | 0.9 | 196 [177.25;219.5] | 188 [170.5;215.5] | 0.5 |
| **HDL**  **(mg/dl)** | 49 [39,25;57] | 46 [38; 55,5] | 0.5 | 47 [37;57] | 42 [37.5;50.5] | 0.5 | 49.5 [40;59] | 47 [39.5;61] | 0.8 |
| **LDL**  **(mg/dl)** | 130 [106:156,75] | 125 [107,75;150,25] | 0.7 | 122.5 [106;161.25] | 125 [107.5;149] | 0.5 | 133 [106;154.25] | 125 [106;155] | 0.8 |
| **AST (UI/L)** | 20 [17;22] | 17,5 [15,25;23,75] | 0.5 | 21 [18;23] | 23.5 [18.5;31.5] | 0.2 | 18 [17;21.25] | 16.5 [14.75;18] | **0.035** |
| **ALT (UI/L)** | 20 [15;23] | 18,5 [14,25;27,25] | 0.7 | 20 [16;25] | 26 [21;37] | **0.027** | 18 [14;23] | 15.5 [11;18.25] | 0.141 |

| **CD4 (n./%)** | 707 [498;930]/36 [28;42] | 695 [532;730]/31.7[25;36] | 0.2/0.6 | 733 [548;1005]/38[28;43] | 695[549;915]/32[24;43] | 0.8/0.3 | 572 [452;816]/35[26;39] | 675[500;741]/30[25;36] | 0.8/0.7 |
| --- | --- | --- | --- | --- | --- | --- | --- | --- | --- |
| **CD8 (n/%)** | 732 [557;963]/36.8[29.6;43] | 864 [708;1084]/39[35;49] | 0.7/0.8 | 722 [559;911]/33.6[28;41] | 884 [663;1078]/37[33;46] | 0.7/0.4 | 741 [553;1028]/40[32;45] | 926[690;1104]/40[34;49] | 0.6/0.3 |
| **Ratio** | 0.9 [0.7;1.35] | 0.8 [0.6;1] | 0.2 | 1.1[0.7;1.6] | 0.8 [0.65;1.25] | **0.03** | 0.8 [0.5;1.2] | 0.8 [0.55;1.05] | 0.71 |
| **Tau (pg/ml)** | 258 [393;176] | 274 [421;185] | 0.6 | 239 [390;146] | 243 [331;188] | 0.1 | 275 [472-214] | 396 [668;169] | 0.5 |
| **pTau (pg/ml)** | 41 [56.5;26.7] | 35  [50;26] | 0.1 | 38  [47;22] | 34  [45;29] | 0.2 | 50 [61;33.5] | 39.7 [58.8-15.8] | **0.018** |
| **Neopterin (ng/ml)** | 0.81 [1.7;0.25] | 1.53 [2.24;0.41] | **0.048** | 0.9 [1.68;0.26] | 1.2 [1.96;0.19] | 0.5 | 0.76 [1.76;0.2] | 1.59 [2.86;0.5] | **0.043** |
| **Beta amyloid 1-42 (pg/ml)** | 919 [1264;729] | 956 [1508;612] | 0.5 | 854 [1213;736] | 950 [1443;863] | 0.09 | 967 [1383;701] | 1149 [1891;395] | 0.7 |
| **CSF NFL (pg/ml)** | 724 [1058;563] | 1055 [1576;471] | **0.001** | 698 [939;510] | 930 [1591;434] | **0.018** | 742 [1097;606] | 1055 [1445;534] | **0.028** |
| **S100Beta (pg/ml)** | 104 [151;55] | 90 [235;59] | 0.5 | 114 [143;63] | 72  [90;43] | 0.3 | 80 [199;50] | 195 [264;90] | 1 |

**Supplementary Table 2.** Markers and variables of interest during the study period, overall and divided by arm. BL: baseline, W24: week 24, SOC: standard of care. Interquantile range [IQR] is provided in brackets. Table describes BL and W24 CSF and biomarkers’ concentrations, both considering the overall population and divided by group. Differences were found in neuromarkers at baseline, pTau resulting higher in the MARAND-X group, and stiffness (resulting higher in the SOC group).

| **Delta CSF cells** | 0 [0;2.5] | 0.5 |
| --- | --- | --- |
| **Delta CSF proteins** | -0.9 [-6;11.6] | 0.5 |
| **Delta CSF glucose** | 2 [-5.2;8.2] | 0.4 |
| **Delta Albumin index** | -0.015 [-0.08;0.08] | 0.3 |
| **Delta IgG index** | 0.015 [-0.04;0.06] | 0.5 |
| **Delta Tau** | 3 [-52;96] | 0.1 |
| **Delta pTau** | -2.95 [-17;3.7] | **0.004** |
| **Delta Neopterin** | 1.1 [-0.6;1.95] | 0.2 |
| **Delta Beta amyloid 42** | 36 [-123;243] | 0.3 |
| **Delta CSF NFL** | 848 [657;1377] | 0.6 |
| **Delta S100 beta** | -23 [-132;68] | 0.7 |
| **Delta stiffness (kPa)** | -0.6 [-.1.8;0.6] | **0.02** |
| **Delta CAP (dB/m)** | -7 [-41;34] | 0.3 |
| **Delta IMT** | 0.03[-0.1;0.12] | 0.7 |
| **Delta APRI** | -0.25[-0.67;0.3] | 0.1 |
| **Delta FIB4** | -0.16 [-0.8;-16] | 0.4 |

**Supplementary** **table 3** Relevant Delta values. Interquantile range [IQR] is provided in bracket.
